# Spatial patterns and spatially-varying factors associated with childhood acute respiratory infection: data from Ethiopian demographic and health surveys (2005, 2011, and 2016)

**DOI:** 10.1186/s12879-023-08273-1

**Published:** 2023-05-05

**Authors:** Solomon Hailemariam Tesfaye, Binyam Tariku Seboka, Daniel Sisay

**Affiliations:** School of Public Health, college of health sciences and medicine, Dilla University, Dilla, Ethiopia

**Keywords:** Acute respiratory infection, Spatial, Patterns, Eigenvector spatial filtering

## Abstract

**Background:**

In Ethiopia, acute respiratory infections (ARIs) are a leading cause of morbidity and mortality among children under five years. Geographically linked data analysis using nationally representative data is crucial to map spatial patterns of ARIs and identify spatially-varying factors of ARI. Therefore, this study aimed to investigate spatial patterns and spatially-varying factors of ARI in Ethiopia.

**Methods:**

Secondary data from the Ethiopian Demographic Health Survey (EDHS) of 2005, 2011, and 2016 were used. Kuldorff’s spatial scan statistic using the Bernoulli model was used to identify spatial clusters with high or low ARI. Hot spot analysis was conducted using *Getis-OrdGi* statistics. Eigenvector spatial filtering regression model was carried out to identify spatial predictors of ARI.

**Results:**

Acute respiratory infection spatially clustered in 2011 and 2016 surveys year (Moran’s *I*:-0.011621–0.334486*)*. The magnitude of ARI decreased from 12.6% (95%, CI: 0.113–0.138) in 2005 to 6.6% (95% CI: 0.055–0.077) in 2016. Across the three surveys, clusters with a high prevalence of ARI were observed in the North part of Ethiopia. The spatial regression analysis revealed that the spatial patterns of ARI was significantly associated with using biomass fuel for cooking and children not initiating breastfeeding within 1-hour of birth. This correlation is strong in the Northern and some areas in the Western part of the country.

**Conclusion:**

Overall there has been a considerable decrease in ARI, but this decline in ARI varied in some regions and districts between surveys. Biomass fuel and early initiation of breastfeeding were independent predictors of ARI. There is a need to prioritize children living in regions and districts with high ARI.

**Supplementary Information:**

The online version contains supplementary material available at 10.1186/s12879-023-08273-1.

## Background

In 2016 there were an estimated 652,572 deaths due to lower respiratory tract infections and ARIs caused 13% of all deaths among children younger than 5 years [1].

Ethiopia ranks 5th among the 15 countries with the highest burden of morbidity and mortality from lower respiratory tract infections among children younger than five years [2]. In Ethiopia, the pooled prevalence of ARIs from 7 studies is 18% [3]. Indoor air pollution from biomass fuel [4, 5], use of an unclean source of energy for cooking [3], malnutrition, tobacco use in the family, maternal literacy [6] and lack of immunization are risk factors for ARIs [7].

Holistic strategies have been developed worldwide by the World Health Organization (WHO) and the United Nations International Children’s Emergency Fund (UNICEF) [8]. This includes; the promotion of exclusive breastfeeding, increase vaccine coverage for measles, full dose of Diphtheria pertussis and tetanus (DPT), *Haemophilus influenza* type b (Hib), and *Pneumococcal conjugate* vaccine (PCV), appropriate care seeking, and antibiotic treatment. Ethiopia has already started implementing this strategy however, the status of the strategy is far beyond the target settled by the WHO [8].

Spatial data analysis is used to provide evidence of a proportion of disease across different geographic areas, identify disease clusters, and assess the impact of potential exposures on disease [9]. Such information enables public health administrators and policymakers in planning. A Previous study from Ethiopia used data from the Ethiopian Demographic and Health Survey (EDHS) of 2016 to assess the spatial distribution of ARIs however, this study didn’t assess the potential impact of spatially varying predictors of ARIs, in addition, the authors did not use data weighing in their analysis [10]. Therefore, this study aimed to explore the spatial patterns and the spatially-varying factors associated with childhood ARI in Ethiopia.

## Methods

### Study setting

Ethiopia is located in the north-eastern segment of the African continent, commonly known as the Horn of Africa, and comprises 1.1 million square km. The country has great geographical diversity, ranging from 4,550 m above sea level to 110 m below sea level. More than half of the country lies above 1,500 m [11]. Ethiopia is the second most populous country in Africa, with a projected population size of 114 million in 2020. Almost half (49.9%) of the population is female, and 15% are children under the age of five years. Most (79%) live in rural communities. Life expectancy at birth is 67.8 years (69.8 years for women and 65.9 years for men), on average [12]. Ethiopia has nine regional states (Afar, Amhara, Benishangul-Gumuz, Gambela, Harari, Oromia, Somali, Southern Nations, Nationalities, and People’s Region (SNNPR), and Tigray) and two administrative cities (Addis Ababa and Dire-Dawa). The regional states and city administrations are divided into zones (as an extended arm of regional states). All the surveys were conducted based on a nationally representative sample for urban and rural areas from the nine regions and two administrative cities.

### Study design and population

Nationally representative population-based data from EDHS, which were conducted in 2005, 2011 and 2016, were used. All the EDHS used two-stage stratified cluster sampling procedures. Each region was stratified into urban and rural areas. In the first stage, a cluster or enumeration areas (EA) were selected with probability proportional to cluster size. Households per cluster were selected systematically in the second stage. The selected EA spatial data (longitude and latitude coordinates) were also included in all three surveys. Detailed study design and settings were described in detail elsewhere [13–15]. All children aged 0 to 59 months living in Ethiopia were considered as the source population. The study population consisted of all children age younger than 5 years in the selected clusters during the EDHS data collection. Data from a total weighted sample of 31,568 children aged younger than 5 years were used for this spatial analysis from the three consecutive EDHS periods (10,109 in 2005, 11,042 in 2011, and 10,417 in 2016).

### Variables of the study

#### Outcome variable

The outcome variable used in this spatial analysis was an acute respiratory infection. ARI is measured using the definition of a child with cough accompanied by [1] short, rapid breathing that is chest related, and/or [2] difficult breathing that is chest related. The data came from the mother’s response to questions about recent episodes of symptoms. Mothers were interviewed if their children had coughs accompanied by short, rapid or difficult breathing.

#### Predictor variables

To reveal potential spatial predictors for the observed hot spot of ARIs, the following predictors were included in the spatial regression model analysis:

##### No occupation

Proportion of mothers not working within the cluster.

##### Biomass fuel

Proportion of households using biomass fuel for cooking.

*Incomplete vaccination*, Proportion of children who started vaccination but missed at least one of the following doses of the vaccine: Bacille Calmette Guerin (BCG), polio, pentavalent, and measles vaccines.

##### Incomplete pneumococcal conjugate vaccine

proportion of children who at least missed one of the three doses of the pneumococcal conjugate vaccine within the cluster.

##### Stunting

Proportion of children with height-for-age less than 2 SD of the median height-for-age, according to WHO international growth criteria (i.e., HAZ < − 2) within the cluster.

##### Wasting

Proportion of children with weight-for-height less than 2 SD of the median height-for-age, according to WHO international growth criteria (i.e., HAZ < − 2) within the cluster.

##### Underweight

Proportion of children with weight-for-age less than 2 SD of the median height-for-age, according to WHO international growth criteria (i.e., HAZ < − 2) within the cluster.

##### Non-exclusive breastfeeding practice

proportion of infants 0–5 months of age who were fed other food or drink, but allow the infant to receive oral rehydration salt, drops, and syrups (vitamins, minerals and medicines) within the cluster.

##### No early initiation of breastfeeding

was defined as the proportion of children aged 0–23 months who did not commence breastfeeding within the first hour of birth.

These key predictors for ARI were selected based on findings from previous studies in Ethiopia [10, 16]. Socio-demographic and economic characteristics of the household were collected during the three surveys. The selected enumeration areas also contain the location (latitude and longitude coordinates). The data set and the Ethiopian district delineation shape file were accessed from the geographic web page of the international DHS programme after registering as an author.

### Data management and analysis

#### Statistical analysis

Data weighting, data cleaning, recording, and descriptive statistics were conducted using STATA version 15. Data were weighted using sampling weight, primary sampling unit, and strata to restore the representativeness of the data and to get more accurate results (estimates, standard errors, and confidence intervals). ArcGIS version 10.8 was used for visualisation, exploration, and maps were created for ARI. SaTscan version 10.0.2 was used to explore significant spatial clusters with ARI and R version 4.1.3 was used for spatial modelling.

#### Spatial analysis

Spatial autocorrelation analysis was used to explore the presence of clustering in the area and detect geographic location of clusters of ARI. The Global Moran’s *I* Index was used to measure the geographic clustering over the study area. Global spatial autocorrelation (Moran’s *I*) is a statistical method used to measure the overall clustering of the data and used to show the strength and pattern of spatial autocorrelation [9]. Moran’s *I* value ranges from-+1 to − 1. Moran’s I values near − 1 showed that the event is dispersed, whereas when Moran’s *I* near + 1 showed that the event is clustered and with a score of zero indicating no clustering. A statistically significant Moran’s *I* (p < 0.05) lead to confirm the existence of spatial autocorrelation [17].

#### Hot spot analysis

Getis-OrdGi* statistics [17] were computed to measure how spatial autocorrelation varies over the study locations by calculating GI* statistics for each area. By comparing local estimates of spatial autocorrelation with global averages, the G*i*(*d*) statistic identifies ‘hot spots’ in spatial data [9]. *Z-*score was calculated to ensure the statistical significance of the ARI clustering at *P-*value < 0.05 associated with 95% CI. The null hypothesis states that the observed clustering of ARI could very likely be the result of a random spatial process. If the *Z*-score is between − 1.96 and + 1.96, the observed clustering of ARI could likely be due to chance. If the score fails outside the range (-1.96 and + 1.96), it is very unlikely that the observed spatial pattern of ARIs is due to a random spatial process. Statistical output with high GI* indicates a hot spot whereas low GI* indicates a cold spot.

#### Spatial scan statistical analysis

Spatial Scan statistical analysis was conducted to test the occurrence of a statistically significant high rate of clusters of ARI using the Bernoulli model by Kuldorff’s SaTScan version 10.0.2. Children who had symptoms of ARI were considered as cases and children who had no symptoms of ARI were considered as controls. We used spatial cluster size < 25% of the population as a higher boundary to detect both large and small clusters. The primary and secondary clusters were identified and the significance of the identified clusters depends on the log-likelihood ratio (*LLR)* test whose P-value is generated using Monte Carlo replications. The *P* value of *LLR* was estimated through 9999 Monte Carlo simulations [9] A p-value less than 0.05 was considered statistically significant. ArcGIS software version 10.8 was used to map the clusters.

#### Spatial regression analysis

We conducted a spatial regression analysis to identify key exposure variables for the observed spatial pattern in ARI using 2016 DHS data. Ordinary least square (OLS), geographically weighted regression (GWR) [18] and eigenvector spatial filtering (ESF) are models used for spatial regression analysis [19]. However, applying the OLS model is uncertain because the model requires spatially autocorrelated residuals, normality of residuals and constant variance [20, 21]. The OLS approach does not incorporate spatial autocorrelation, indicating a misspecification error [19, 21]. The ESF is a regression model to estimate regression coefficients in the presence of spatial dependence and it is becoming a popular approach to assess spatial variation in outcome variables [19]. The ESF regression model effectively removes autocorrelated residuals, reduces spatial misspecification errors, increases the strength of the model fit, increases the normality of model residuals, and increases the homoscedasticity of model residuals [21], it effectively handles spatial dependencies [22]. For this study, we used the random effect ESF (RE-ESF) regression model which is an extension of the ESF model using the *spmoran* package in R version 4.1.3 [23]. The RE-ESF estimates regression coefficients more accurately and reduces errors than ESF [22].

In addition, the RE-ESF based spatially varying coefficient model also known as Moran spatially varying coefficient (M-SVC) was used to model spatially varying relations. The M-SVC is a local form of regression which outperforms the GWR, which is another SVC estimation approach, in terms of coefficient estimates accuracy, and computational time [23].

## Results

### Sample characteristics

A weighted sample of 31, 568 children under the age of 5 years were included for spatial analysis of ARI. Table 1 shows the characteristics of mothers and children across different variables. The majority of children, 9357 (92.6%) in 2005, 9606 (87%) in 2011, and 9254 (88.8%) in 2016 were from rural areas. In 2005 EDHS more than three-fourths of mothers were not educated. Regarding the type of fuel for cooking, about 30,980 (98%) of the households used biomass fuel for cooking.

**Table 1:** Characteristics of mothers and children under five years of age, in Ethiopia

Variables	EDHS		
	2005 n (%)	2011 n (%)	2016 n (%)
Sex			
Male	5129 (50.7)	5676 (51.4)	5342 (51.3)
Female	4980 (49.3)	5366 (48.6)	5075 (48.7)
Age in month			
< 6	582 (11.9)	1261 (11.7)	1200 (11.5)
6 - 11	502 (10.2)	1118 (10.4)	1071 (10.3)
12 - 23	922 (18.8)	1904 (17.7)	2004 (19.2)
24 - 35	927 (18.9)	2000 (18.6)	1944 (18.7)
36 - 47	1030 (31.0)	2302 (21.4)	2007 (19.3)
48 - 59	948 (19.3)	2177 (20.2)	2191 (21.0)
Residence			
Rural	9357 (92.6)	9606 (87.0)	9254 (88.8)
Urban	752 (7.4)	1436 (13.0)	1163 (11.2)
Women’s education level			
No education	7951 (78.7)	7610 (68.9)	6858 (65.8)
Primary	1709 (16.9)	3012 (27.3)	2806 (26.9)
Secondary	407 (4.0)	252 (2.3)	493 (4.7)
Higher	42 (0.4)	168 (1.3)	260 (2.5)
Wealth quintile			
Poorest	2218 (21.9)	2476 (22.4)	2499 (24.0)
Poorer	2122 (21.0)	2444 (22.1)	2386 (22.9)
Middle	2210 (21.9)	2277 (20.6)	2159 (20.7)
Rich	2015 (19.3)	2158 (19.4)	1860 (17.9)
Richest	1544 (15.3)	1687 (15.3)	1513 (14.5)
Women’s occupation level			
Working	2967 (29.4)	5921 (53.6)	4624 (44.4)
Not working	7142 (70.6)	5121 (46.4)	5793 (55.6)
Type of cooking fuel			
Electricity	7 (0.1)	47 (0.4)	297 (2.9)
Liquid petro gas	5 (0.1)	8 (0.1)	38 (0.4)
Kerosene	124 (1.2)	55 (0.5)	7 (0.1)
Biomass fuel	9973 (99)	10,932 (99.0)	10,075 (96.7)
Region			
Tigray	653 (6.5)	702 (6.4)	686 (6.6)
Afar	96 (1.0)	111 (1.0)	105 (1.0)
Amhara	2312 (22.9)	2478 (22.4)	1967 (18.9)
Oromia	4017 (39.7)	4665 (42.3)	4571 (43.9)
Somali	432 (4.3)	339 (3.1)	476 (4.6)
Benishangul-Gumuz	95 (1.0)	127 (1.2)	113 (1.1)
SNNPR	2273 (22.5)	2305 (20.9)	2169 (20.8)
Gambella	29 (0.3)	37 (0.3)	25 (0.2)
Harari	22 (0.2)	27 (0.2)	24 (0.2)
Addis Ababa	146 (1.4)	214 (1.9)	237 (2.3)
Dire-Dawa	34 (0.3)	37 (0.3)	44 (0.4)

### Overall spatial patterns of ARI

Overall, childhood ARI shows decreasing patterns in Ethiopia. The proportion of children with ARI was 12.6% (95%, CI: 0.113–0.138) in 2005, 7.1% (95%, CI: 0.061–0.079) in 2011, and 6.6% (95% CI: 0.055–0.077) in 2016. Between 2005 and 2016 the prevalence of symptoms of ARI decreased by 47.6% (from12.6% in 2005 to 6.6% in 2016). The spatial patterns of ARI in Ethiopia were clustered in the 2011 and 2016 study periods. The spatial pattern of ARI in 2005 does not appear to be significantly different than random (global Moran’s *I* value − 0.011621, P-value = 0.798). The spatial autocorrelation analysis revealed that the global Moran’s *I* values and *Z-*Scores were positive for the 2011 and 2016 survey periods indicating the presence of statistically significant clusters (Table 2).

**Table 2:** Summary of spatial autocorrelation of ARI in Ethiopia

EDHS study period	Observed Moran’s *I*	Expected Moran’s *I*	*Z-*Score	*P*- Value
2005	-0.011621	-0.001946	-0.255710	0.798
2011	0.334486	-0.001754	10.639772	< 0.001
2016	0.091175	-0.001610	3.076921	0.002

### The spatial patterns of ARI at the regional level

**Fig. 1:**
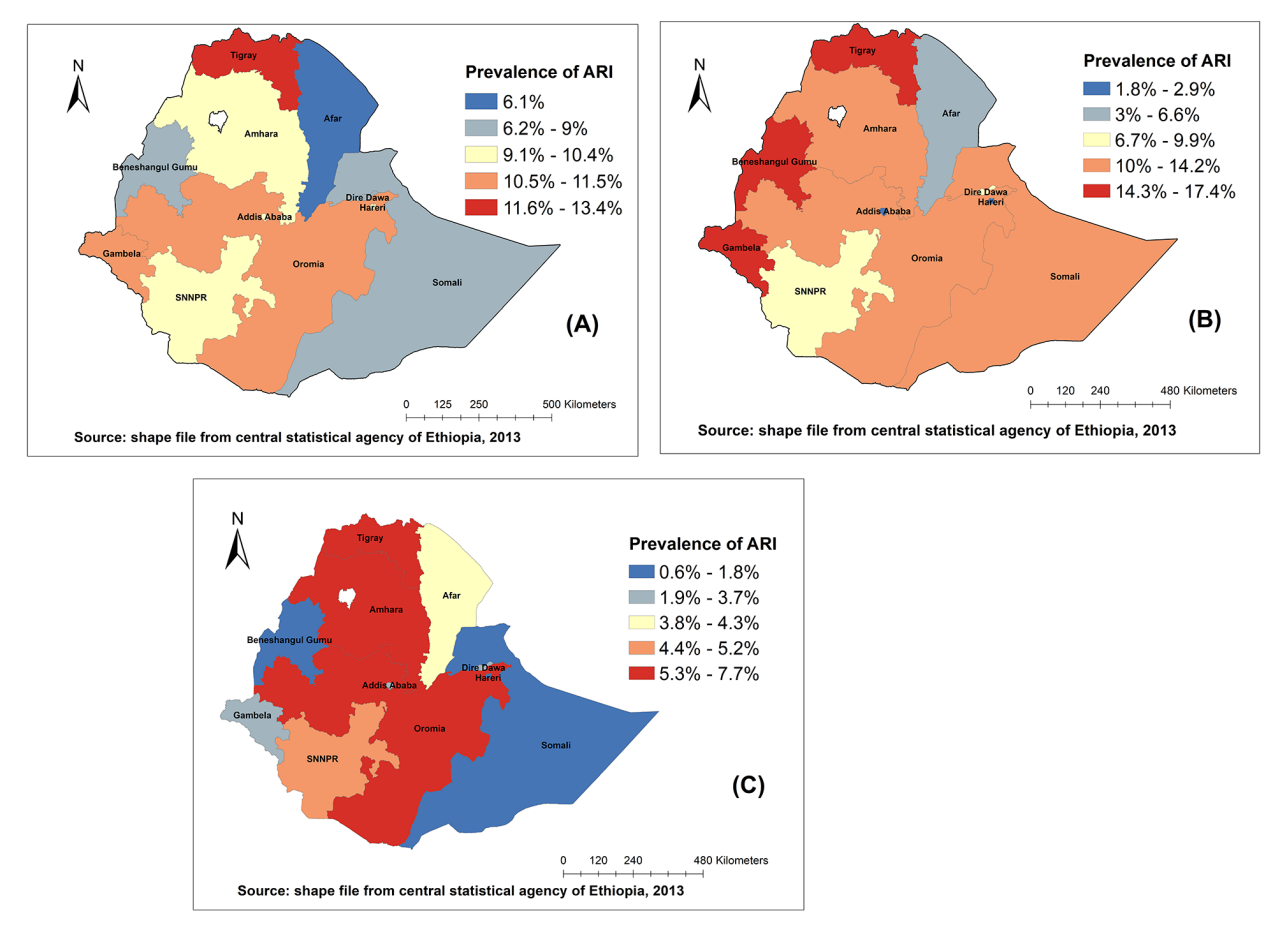
Spatial patterns of childhood ARI at the regional level in Ethiopia: 2005 (A), 2011 (B), 2016 (C)

Figure 1 shows the spatial patterns of ARI by region in Ethiopia. In 2005, the highest proportion of ARI ranging from 11.6 − 13.4% was observed in the Tigray region followed by 10.5 − 11.5% in Oromia and Gambella regions. While the lowest prevalence was seen in the Afar region (6.1%) (Fig. 1A). In 2011 the prevalence of ARI increased in Benishangul-Gumuz, Gambella, and Amhara regions. There was also an increment in Somali and Afar regions and the prevalence remains high in the Tigray region (Fig. 1B). Tigray, Amhara, and Oromia regions had the highest prevalence of ARI (from 5.3 − 7.7%), while the prevalence decreased in Somali region in 2016 (Fig. 1C).We produce map of standard deviation classifications for prevalence of ARI to show regions that have prevalence of ARI below or above the average national prevalence of ARI. In 2005, Tigray, Oromia, Benishangul-Gumz, and Gambella regions have prevalence of ARI above the average prevalence of ARI. While, Afar region has prevalence of ARI below the average prevalence of ARI. Additional figure file shows this in more detail [see additional file 1].

### The spatial patterns of ARI at the Zonal (district) level

**Fig. 2:**
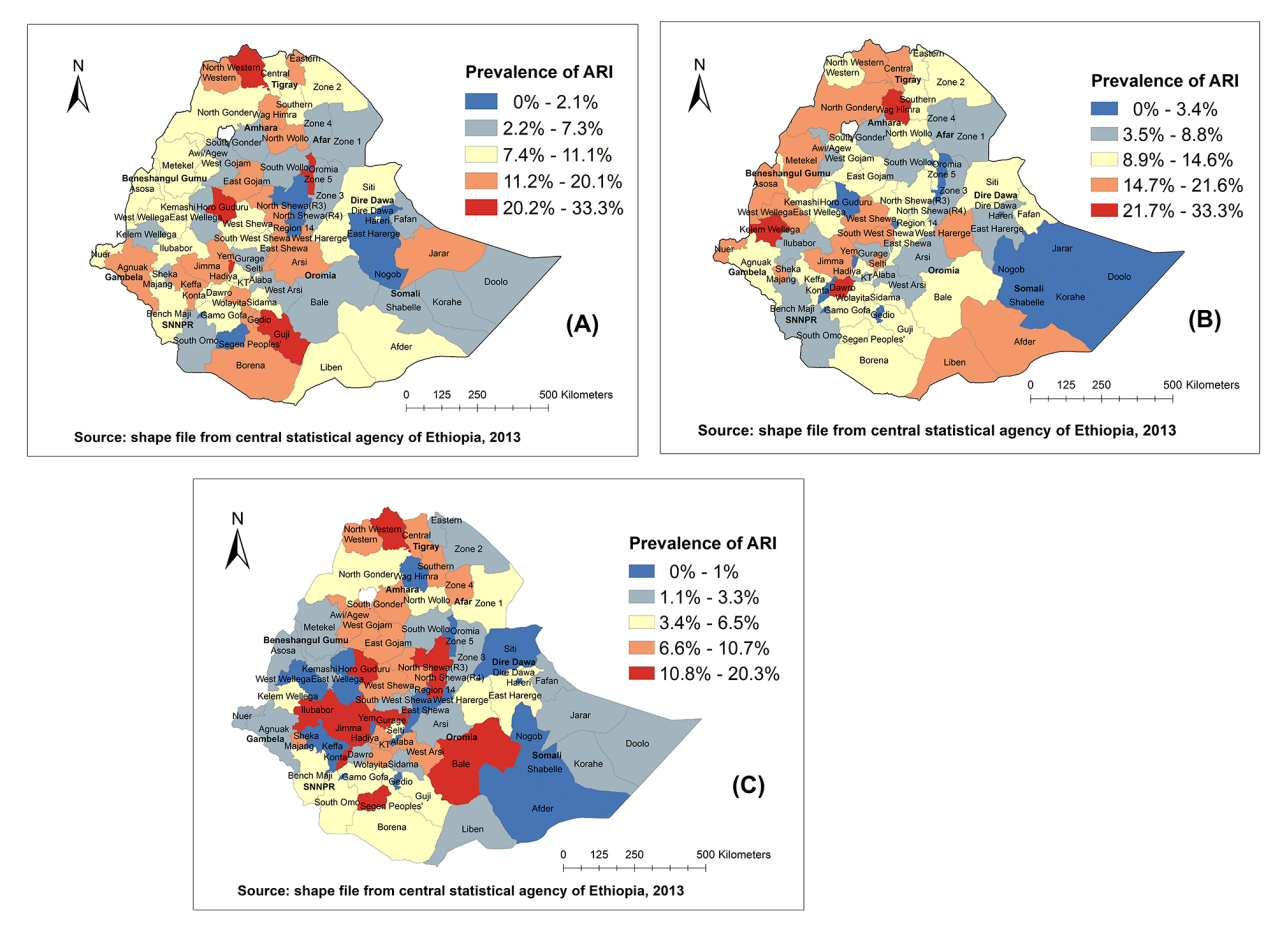
Spatial patterns of childhood ARI at zone/district level in Ethiopia: 2005 (A), 2011 (B), 2016 (C)

The spatial patterns of ARI revealed the presence of uneven patterns across Ethiopia zones. There are zones/ districts with high prevalence (red colour) of ARI in Ethiopia across the survey periods (Fig. 2). Additional figure shows districts with prevalence of ARI above and below the national average prevalence of ARI [see additional file 2].

### Hot spot and cold spot areas of ARI

**Fig. 3:**
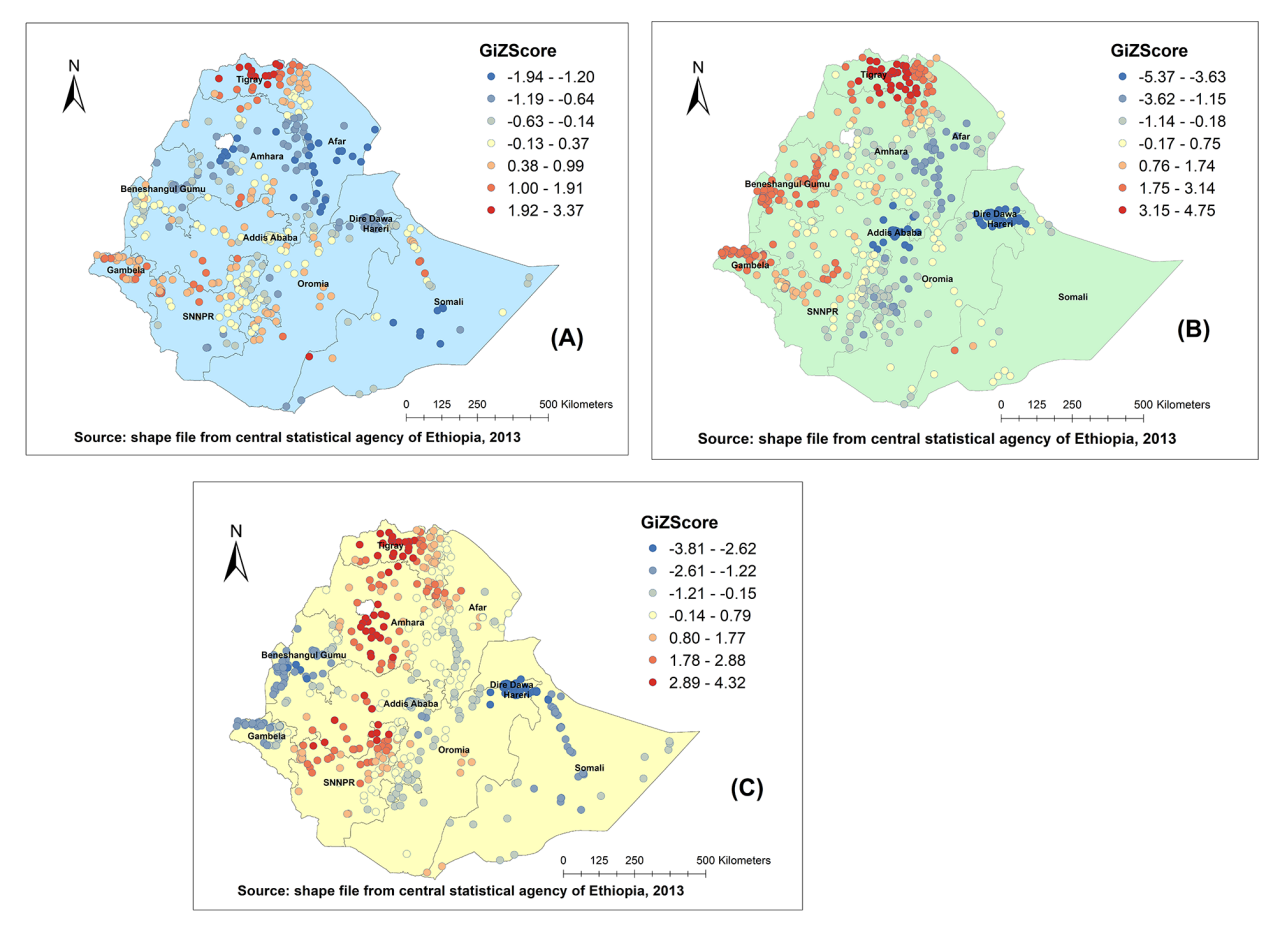
Hot spot and cold spot identification of childhood ARI in Ethiopia, 2005 (A), 2011 (B), 2016 (C). The red colour indicates high hot spot (high risk) areas for ARI, the blue colour indicates cold spot (low risk) areas for ARI

The Getis–Ord analysis identified hot spots (high-risk areas) and cold spots (low-risk areas). Figure 3 shows the hot spot and cold spot areas for ARI. There were consistently hot spot zones in the northern part of the country in all the survey periods. In 2005 Western and central zones of Tigray had the highest risk (Fig. 3A). North Western and central part of the Tigray region, North Gondar, Agew/Awi, Wag himra, zones of Amhara region, and Nuer zones of Gambella region, and Jimma zone of Oromia region were significant hot spot areas, To the contrary, East and south-west Shewa, and East Harerge zones of Oromia region were cold spot for ARI in 2011 (Fig. 3B). In 2016 most of the hot spot zones were located in Western, North Western, and Central zones of Tigray region, North Gondar, South Gondar, West and East Gojam zones of Amhara region, Horo-Guduru, Illiubabur, Jimma, zones of Oromia region, and Keffa zone of SNNPR region. On the other hand, the Fafan zone of the Somali region, East Harerge zone of the Oromia region Asosa zone of Benishangul-Gumuz and Nuer zone of Gambella were detected as cold spots for ARI (Fig. 3C).

### Spatial scan statistical analysis

**Fig. 4:**
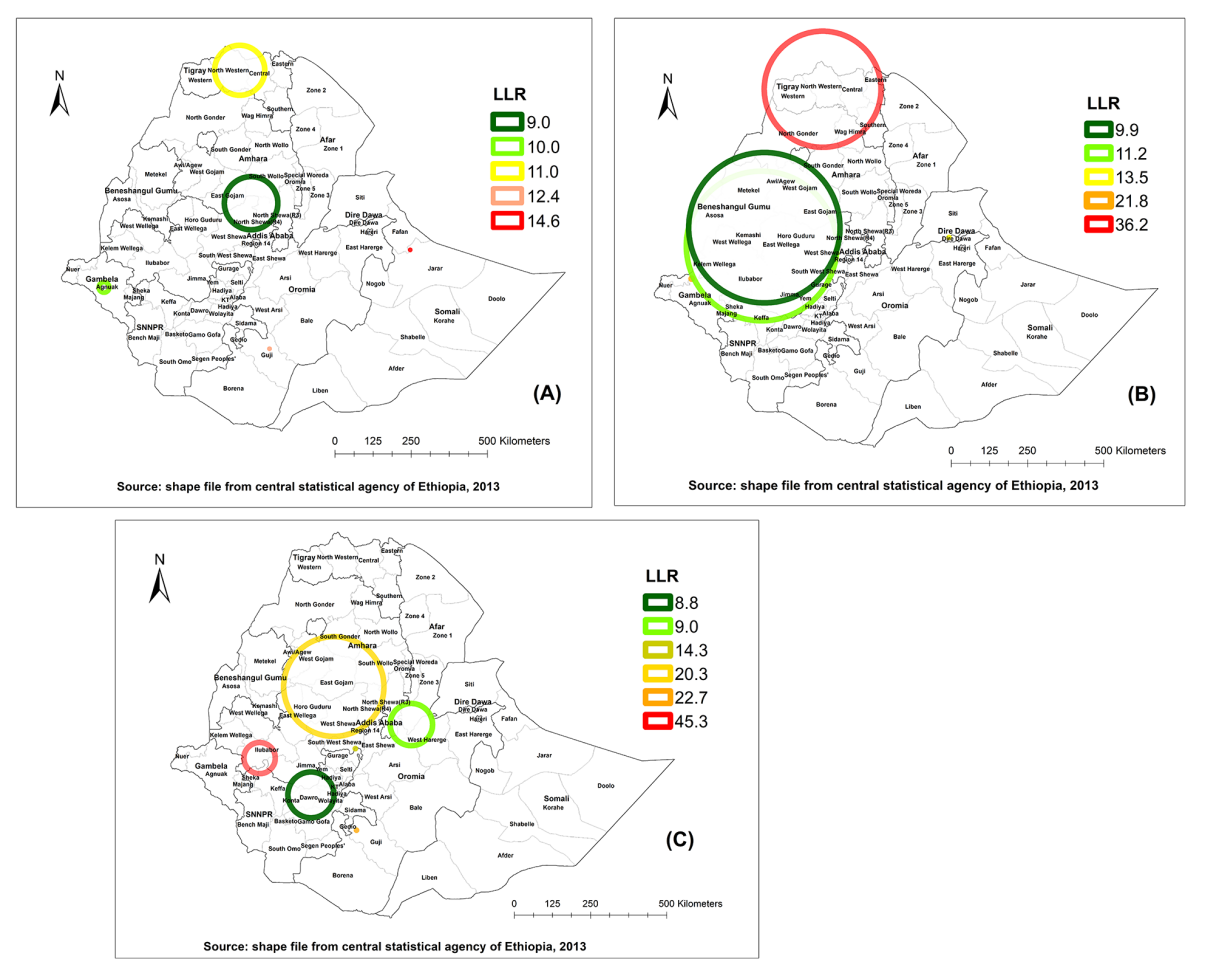
Spatial clustering of childhood ARI in Ethiopia: 2005 (A), 2011 (B), 2016 (C)

In 2005 the primary cluster for ARI (LLR = 14.6, P < 0.001was centred in the Jarar zone of the Somali region (8.751617 N, 43.379093 E) and relative risk of 4.5 (Table 3; Fig. 4A). This suggests that children who lived in this zone were 4.5 times more likely to have ARI compared to children living outside of these clusters. The cluster spatial window in 2011 EDHS was centred at 14.013880 N, 37.983067 E, with a radius of 191.27 km, relative risk 1.87 and LLR = 36.2, P < 0.001. These primary clusters incorporate all zones of Tigray region (North Western, Western, Central, Eastern, and Southern), North Gondar, and.

**Table 3:** Significant clusters of ARI in Ethiopia, 2005, 2011, and 2016 EDHS

Year	Detected Clusters	(Coordinate)/radius in Km	Population	Cases	RR	LLR	P-value
2005	Primary	(8.751617 N, 43.379093 E) /0	33	16	4.49	14.6	< 0.001
	Secondary	(5.844300 N, 39.182880 E) / 0	13	9	6.37	12.4	< 0.001
	Secondary	(14.101627 N, 38.282906 E) / 82.27	266	55	1.94	11.0	0.011
	Secondary	(7.638066 N, 34.266033 E) / 17.22	68	21	2.86	10.0	0.021
	Secondary	(10.160658 N, 38.634846 E) / 87.57	204	43	1.97	9.0	0.041
2011	Primary	(14.013880 N, 37.983067 E) / 191.27	1161	228	1.87	36.2	< 0.001
	Secondary	(8.367302 N, 34.102268 E) / 0	20	15	6.56	21.8	< 0.001
	Secondary	(9.608009 N, 41.777573 E ) / 0	17	11	5.64	13.5	< 0.001
	Secondary	(9.356925 N, 36.180122 E ) / 246.32	1979	291	1.36	11.2	0.01
	Secondary	(9.898865 N, 36.257153 E ) / 247.59	1755	259	1.35	9.9	0.022
2016	Primary	(8.130989 N, 35.637974 E) / 51.36	87	37	6.72	45.3	< 0.001
	Secondary	(6.005498 N, 38.525295 E) / 0	17	12	10.81	22.7	< 0.001
	Secondary	(10.265456 N, 37.841267 E) / 164.32	1173	133	1.88	20.3	< 0.001
	Secondary	(8.425886 N, 38.488144 E) / 0	39	14	5.50	14.3	< 0.001
	Secondary	(9.138033 N, 40.159630 E) / 69.52	309	41	2.06	9.0	0.034
	Secondary	(7.050249 N, 37.171494 E) / 75.44	604	67	1.75	8.8	0.037

Wag Himra zones of the Amhara region. The secondary cluster centred at 8.367302 N, 34.102268 E, with a relative risk of 6.56 was detected in the Nuer zone of the Gambella region (Table 3; Fig. 4B). The primary cluster for ARI (LLR = 45.3, P < 0.001) was centred at 8.130989 N, 35.637974 E about 51.36 km radius with a relative risk of 6.72. It surrounds the Ilubabor, and Jimma zone of the Oromia region, and the Sheka and the Keffa zones of SNNPR (Table 3; Fig. 4C).

### Spatial predictors of ARI

The spatial regression analysis showed that those households who used biomass fuel for cooking and children who didn’t commence breastfeeding within the first hour of birth were associated with spatial variations in childhood ARI (Table 4). About 20.5% of the spatial variability in ARI was explained by the model (adjusted R^2^ = 0.205). We performed model validation tests between OLS, GWR, MGWR, and RE-ESF models, the same predictors were used and Akaike information criterion (AIC), Bayesian information criterion (BIC), and adjusted R-square were used for comparing the model validation. Overall the RE-ESF model outperformed OLS, GWR, and MGWR models detail results are presented in additional file (see additional file 3).

**Table 4:** Factors associated with spatial variations of ARI using 2016 EDHS

Exposure variables	Estimate	Standard error	*t-*value	P-value
Intercept	0.072	0.023	3.157	0.002
Households using biomass fuel	0.050	0.024	2.068	0.039
Women with no occupation	-0.033	0.018	-1.864	0.063
Children with incomplete Pneumococcal conjugate vaccine	-0.029	0.023	-1.277	0.202
Children with incomplete pentavalent vaccine	-0.000	0.000	-0.952	0.341
Children with stunting	-0.009	0.037	-0.257	0.797
Children with under-weight	-0.045	0.038	-1.200	0.230
Children with wasting	0.030	0.047	0.644	0.519
No exclusive breastfeeding	0.023	0.031	0.743	0.458
No early initiation of breastfeeding	0.047	0.021	2.239	0.026

**Fig. 5:**
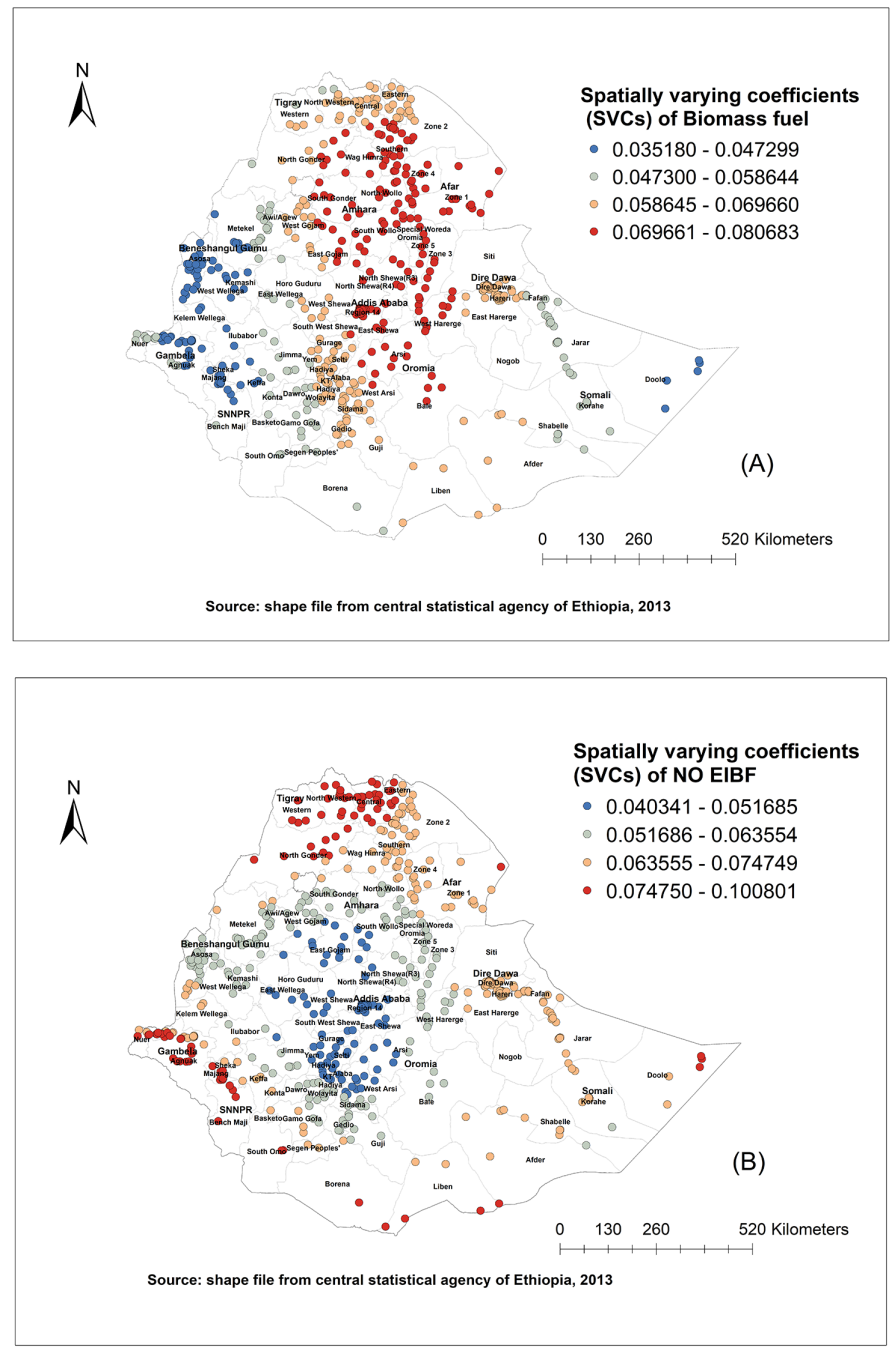
Spatial variations of ARI with biomass fuel use (A), EIBF (B), 2016 EDHS

The M-SVC regression model showed that households who used biomass fuel for cooking and children not commencing early breastfeeding were positively associated throughout the clusters (Fig. 5). The strength of the association between biomass fuels (Fig. 5A) and being not initiated early breastfeeding with childhood ARI varies across the clusters. The impact of not initiated early breastfeeding varies across the cluster and is a strong predictor in all parts of Tigray region, North Gondar of Amhara region, in some parts of Gambella and SNNPR regions (Fig. 5B). Coefficients of other exposure variables were held constant. The M-SVC model has explained about 22% of the spatial variations in childhood ARI. The average standard error was 0.021 and 0.031 for biomass fuel and EIBF, respectively. Map of standard errors of the spatially varying coefficients of biomass fuel and early breastfeeding initiation from M-SVC model was presented as additional file (see additional file 4).

## Discussion

There has been a considerable decrease in ARI in children younger than five years in Ethiopia between 2005 and 2016. However, this reduction has not been seen similar across the study locations. There were consistent hot spot areas in the Northern part of the country. Clusters, where a large proportion of households used biomass fuel for cooking and a large proportion of children did not commence breastfeeding immediately one hour after birth; have a positive association with ARI in much of Northern and some of the Western parts of the country.

Our results suggest that the overall spatial patterns of ARI in children younger than 5 years have significantly decreased by about 48% between 2005 and 2016. Likewise, globally there is a decline in respiratory infections by 21.8% between 1990 and 2016 among children younger than five years [1]. The study from the Zambian demographic and health survey also shows a decline in ARI between 1996 and 2014 [24].There are a number of possible explanations for the decline in ARI observed globally, including improvements in socio-economic status, child nutrition, Human Immunodeficiency Virus (HIV) control, improved case management of ARI, and availability of vaccines [25].

Global Action Plan for the Prevention of Pneumonia and Diarrhoea (GAPPD) is a strategy recommended by World Health Organization aiming to reduce childhood death due to pneumonia and Diarrhoea [26]. The GAPPD is an integrated approach that ensures every child has access to proven and appropriate preventive and treatment measures for ARI. The GAPPD strategies include promoting: [1] exclusive breastfeeding and adequate complementary feeding to protect children from pneumonia; [2] vaccination; [3] hand hygiene; [4] reductions in household air pollution; [5] HIV prevention; [6] co-trimoxazole prophylaxis for HIV infected and exposed children; and [7] treatment of childhood pneumonia with antibiotics and oxygen. Ethiopia is implementing this strategy since 2015 and the overall achievement of the GAPPD is improving.[8] However, GAPPD implementation showed that Ethiopia is far from reaching the target set by WHO [26]. Ethiopia has made impressive gains in child survival related to ARI through the implementation and scale-up of IMNCI and community case management of ARI [27].

Even though the magnitude of ARIs was decreasing in Ethiopia, the decline in spatial patterns of ARI is uneven. For example, in 2016 the prevalence of ARI ranges from 1 to 20% in some districts of the country. This variation in spatial patterns of ARIs was evidenced by Moran’s *I* statistics which show strong clustering of ARI and the hot spot analysis which detected consistently districts in the Northern part of Ethiopia in all three surveys and the central part of the country in the 2016 survey.

Direct comparisons of the spatial variations and the impact of risk factors on ARI between studies are difficult because of differences in definitions of ARI, analysis methods, and study participants. However, the existence of spatial variations in hospitalization due to pneumonia has been noted among children younger than 5 years hospitalized for severe pneumonia in Brazil [28] and Bhutan [29]. Longitudinal data from Ethiopia between 2009 and 2017 shows that ARI is still a major cause of death in under five years children [30]. Therefore, Ethiopia needs to strengthen the GAPPD strategy to overcome the spatial variations observed in ARI across the study districts.

The eigenvector spatial filtering model reveals that the spatial variations in ARI among children age younger than 5 years were likely to be affected by the majority of households that use biomass fuel for cooking and the majority of children who did not commence breastfeeding immediately after birth. In addition, the M-SVC model showed these two risk factors have a strong impact on acquiring ARI. A Previous study from Ethiopia indicated that biomass fuel has been associated with ARI among children younger than five years. [4] Meta-analysis of 8 studies from Ethiopia found that child holding during cooking and using unclean sources of energy for cooking are significant predictors of ARI [3]. Data from Zambia’s demographic and health surveys show using charcoal or wood for cooking increases the risk of developing ARI as compared to those who use electricity [24]. In Ethiopia, biomass fuel is burnt in open fires indoors. The burning of biomass fuel in unventilated indoors, results in a significant concentrations of hazardous pollutants, carbon monoxide, particulate matter, nitrogen oxide and polyaromatic hydrocarbons, leading to household air pollution and children experiencing high exposure [31]. Therefore, a rapid increase in the use of modern biomass technology to ensure clean and efficient use of energy needs to be a priority agenda for policymakers.

Children who were breastfed within 1-hour of birth had a statistically significant association with ARI and the effect spatially varies across the geographic location. The effect is strong in many of the Northern, some parts of the Southern, and some Western parts of the country. Our spatial analysis did not find any evidence of an association between exclusive breastfeeding and ARI. However, evidence from Ethiopia shows both early initiations of breastfeeding and exclusive breastfeeding are associated with a lower risk of ARI [16] and evidence from Zambia indicated that exclusive breastfeeding is a significant predictor of ARI [24]. The first breast milk contains colostrum, which is highly nutritious and has antibodies that provide immunity to the child and assist in the maturation of the child’s immune system to protect the newborn from infections [32]. We were not able to show an association between exclusive breastfeeding and ARI. Similarly, mother-child cohort study failed to show an association between the duration of breastfeeding and the longitudinal pattern of respiratory infection, but the study show duration of breastfeeding has a protective effect only throughout the first year of life [33].

Previous research has indicated that wasting [6], stunting [3], and being underweight are predictors of ARI [24]. The current spatial study showed that the risk of ARI in areas with a large proportion of wasting, stunting, and underweight was not statistically significant from what were in clusters with low a proportion of wasting, stunting, and underweight. Evidence suggests that increased risk of ARI in children depends on multiple risk factors, including, lack of vaccines [34], malnutrition [6] using unclean energy for cooking [4, 5], breastfeeding [16], overcrowding [35], low birth weight [36], and the existence of HIV [37]. The lack of association between malnutrition and ARI might be explained by the fact that to develop ARI multiple risk factors must be present. Therefore, in our analysis, we lack control for some of these risk factors (potential confounders).

We did not observe evidence of a positive association between a low proportion of vaccine coverage and a high risk of ARI as expected. On the contrary, we observe a statistically non-significant negative association between vaccines and the risk of ARI. This result should be interpreted cautiously. Pneumococcal conjugate vaccines and Hib vaccines are known to effectively prevent ARI due to *streptococcus Pneumoniae* and Hib [34]. However, in our study vaccines were not significant determinants of ARI at the cluster level. The following are possible explanations for this observed non-significant association. First; it may be a consequence of district-level aggregation of vaccines. Second; viral and atypical bacteria are common causes of ARI [38]. A study from Morocco has shown that viruses are the predominant cause of ARIs in preschool children [39]. Even though vaccines have played important role in preventing bacterial respiratory infections, there are non-vaccine pneumococcal serotypes responsible for ARIs [40]. The negative association between vaccines and ARI might indicate that more children have ARI in areas where vaccine coverage is high, as compared to children living in areas where vaccine coverage is low. With this regard, there is a need to conduct further epidemiological studies for identifying aetiologies of ARI, particularly among vaccinated children.

We used data collected from nationally representative large geographic areas that enable the findings to be generalized across the country. This spatial study provides evidence of the important areas concerning ARI in children and its association with exposure variables, which supports public policies to reduce child mortality for the sustainable development goal.

As a limitation, we utilized secondary data as such some known risk factors of ARI were not assessed. The outcome variable ARI was measured based on self-report from the mother leading to recall bias. The newest collected data was 6 years at the time of analysis. However, the results of this study are relevant for understanding the spatial variations of childhood ARI and its spatial predictors in geospatial areas.

## Conclusions

Even though the magnitude of ARI in children younger than 5 years was declining between 2005 and 2016, there was significant variation in spatial patterns of ARI across the geographic areas. There were consistent hot spot areas in the Northern part of Ethiopia throughout the three waves of EDHS. Uses of biomass fuel for cooking and not initiating breastfeeding within 1-hour of the birth of the baby have significant effects on acquiring ARI. Consistent with the hot spot analysis the effect of these two variables were observed to be strong in the Northern and some Western part of the country. Ethiopia needs to strengthen the GAPPD strategy to achieve a similar decline in ARI across different geographic areas. In addition, there is a need for region and district-specific policies that focus on the identified risk factors. As we observed that maternal occupation and stunting, underweight, and vaccination status of a child were negatively associated with ARI, there is a need to gain a more detailed understanding of these potential risk factors of ARI.

## Electronic supplementary material

Below is the link to the electronic supplementary material.

Supplementary Material 1

Supplementary Material 2

Supplementary Material 3

Supplementary Material 4

## Data Availability

The datasets generated and/or analyzed during the current study are available from DHS website (www.dhsprogram.com).

## List of Abbreviations

ARI: Acute Respiratory Infection
BCG: Bacille Calmette Guerin
DPT: Diphtheria, Pertussis and Tetanus
EA: Enumeration Areas
EDHS: Ethiopian Demographic Health Survey
ESF: Eigenvector Spatial Filtering
GAPPD: Global Action Plan for the Prevention of Pneumonia and Diarrhoea
GWR: Geographically Weighted Regression
Hib: *Haemophilus influenza* type b
HIV: Human Immunodeficiency Virus
IMNCI: Integrated Management of Childhood Illness
LLR: Log-Likely hood Ratio
M-SVC: Moran spatially varying coefficient
OLS: Ordinary Least Square
PCV: *Pneumococcal conjugate* vaccine
RE-ESF: Random Effect Eigenvector Spatial Filtering
SNNPR: Southern Nations, Nationalities, and People’s Region
SD: Standard Deviation
UNICEF: United Nations International Children’s Emergency Fund
WHO: World Health Organization
