# Supplementary material for: Spatial patterns and spatially-varying factors associated with childhood acute respiratory infection: data from Ethiopian demographic and health surveys (2005, 2011, and 2016)
Source: BMC Infect Dis. 2023 May 5;23:293. doi: 10.1186/s12879-023-08273-1 (PMC10163815; doi:10.1186/s12879-023-08273-1)
Supplement: Supplementary file 3 — Supplementary Material 3 [file 12879_2023_8273_MOESM3_ESM.pdf]

Additional file 3: Comparison of performance between spatial regression models

Supplementary table 1: validation test between the OLS, GWR, MGWR, and RE-ESF models

Outcome measures	OLS	GWR	MGWR	RE-ESF
AIC	4728.994	1722.741	4687.556	-966.69121146
BIC	-	1925.255	4790.226	-891.33122989
Adjusted R square	0.042	0.131	0.122	0.22030476
